# Gene Expression and Regulation in Adrenocortical Tumorigenesis

**DOI:** 10.3390/biology2010026

**Published:** 2012-12-27

**Authors:** Annabelle L. Fonseca, James Healy, John W. Kunstman, Reju Korah, Tobias Carling

**Affiliations:** 1 Department of Surgery, Yale Endocrine Neoplasia Laboratory, Yale University School of Medicine, 333 Cedar Street, TMP202 Box 208062, New Haven, CT 06520, USA; E-Mails: james.healy@yale.edu (J.H.); john.kunstman@yale.edu (J.W.K.); tobias.carling@yale.edu (T.C.); 2 Yale Endocrine Neoplasia Laboratory, Yale University School of Medicine, New Haven, CT 06520, USA; E-Mail: reju.korah@yale.edu

**Keywords:** adrenocortical tumor, epigenetics, gene expression, genetics

## Abstract

Adrenocortical tumors are frequently found in the general population, and may be benign adrenocortical adenomas or malignant adrenocortical carcinomas. Unfortunately the clinical, biochemical and histopathological distinction between benign and malignant adrenocortical tumors may be difficult in the absence of widely invasive or metastatic disease, and hence attention has turned towards a search for molecular markers. The study of rare genetic diseases that are associated with the development of adrenocortical carcinomas has contributed to our understanding of adrenocortical tumorigenesis. In addition, comprehensive genomic hybridization, methylation profiling, and genome wide mRNA and miRNA profiling have led to improvements in our understanding, as well as demonstrated several genes and pathways that may serve as diagnostic or prognostic markers.

## 1. Introduction

Adrenocortical tumors are frequently found in the general population, with an estimated prevalence of 3%–7% [1,2] and are most often discovered incidentally [3]. These tumors may be, more commonly, benign adrenocortical adenomas (ACA). Malignant adrenocortical carcinoma (ACC) is a rare malignancy with an estimated incidence of 0.7–2.0 cases per million per year [2,3,4].

Unfortunately the clinical, biochemical and histopathological distinction between ACA and ACC may be difficult in the absence of widely invasive or metastatic disease [5,6,7,8,9,10]. At present the *Modified Weiss Score* (a histopathologic scoring) is the most widely accepted means of classifying ACC. However, there is significant variation in inter-observer agreement and it does not identify molecular targets for diagnosis and therapeutic intervention. A better method of distinguishing benign from malignant ACTs is therefore needed, and attention has turned towards a search for molecular markers [11]. Current understanding of the development of adrenocortical carcinoma in the last decade originated, in large part, due to the study of rare genetic diseases that are associated with the development of ACC [12]. In addition, comprehensive genomic hybridization, methylation profiling, and genome wide mRNA and miRNA profiling have, in the more recent past, led to improvements in our understanding, as well as demonstrated several genes and pathways that may serve as diagnostic or prognostic markers [13,14,15,16,17].

### 1.1. Genetic Predisposition to Adrenocortical Tumors and Hyperplasias

A number of hereditary syndromes are associated with the development of ACT and hyperplasias, and have contributed to our understanding of adrenocortical tumorigenesis. Mutations responsible for a number of familial cancer syndromes (Beckwith-Wiedemann syndrome, Li-Fraumeni syndrome, Multiple Endocrine Neoplasia type 1) have also been shown to present as somatic mutations in sporadic ACC.

### 1.2. Li-Fraumeni Syndrome

Li-Fraumeni syndrome is an autosomal dominant cancer predisposition syndrome that results from a germline mutation in the *TP53* gene located on chromosome 17p13. *TP53* controls cell growth by induction of target genes that arrest the cell cycle in the G1-S phase and induce apoptosis [18]. Germline *TP53* mutations have been identified in 70% of families with Li-Fraumeni syndrome [19]. ACC develops in 3%–4% of patients with this syndrome [11]. Germline TP53 mutations have also been observed in 50%–80% of children and 30%–35% of adults with apparent sporadic ACC [20,21,22,23]. However, a recent study has demonstrated that TP53 germline mutations may be rare in adult patients, and approximates a prevalence of 13% in patients under 40 years old with ACC [24]. Inactivating somatic mutations in TP53 have also been observed in sporadic ACC in exons 5–8 and exons 2–11 [11]. A specific germline mutation (Arg337His) located on exon 10, affecting residues on the *C*-terminal domain of the p53 protein responsible for oligomerization was identified in 77%–97% of children with benign or malignant sporadic ACT and 13.5% of adults with sporadic ACT in southern Brazil, and was associated with an unfavorable prognosis in adults, but not in children with ACT [25,26]. Loss of heterozygosity (LOH) at 17p13 has been demonstrated to occur in 85% of ACC and 30% of ACA, and has been implicated as a marker of recurrence after resection in addition to correlating with the *Modified Weiss Score* [27]. The presence of inactivating *TP53* mutations in ACC was associated with worse prognosis on transcriptome analysis [28].

### 1.3. Beckwith-Wiedemann Syndrome

Beckwith-Wiedemann syndrome occurs in an inherited fashion in 15% of cases [11]. The *IGF-2* gene, located at chromosome 11p15, is a potent mitogen, and encodes a fetal growth factor that is maternally imprinted and expressed only from the paternal allele [29]. Defects in the imprinted 11p15 region increase IGF-2 expression in patients with Beckwith-Wiedemann syndrome. Overexpression of IGF-2 in observed in the majority of ACCs [30,31,32]. Furthermore, LOH at 11p15 has been shown to occur in 78% of ACC and 9% of ACA, and correlates with a higher risk of recurrence [27]. Transcriptome analysis has demonstrated that *IGF-2* is the gene most overexpressed in ACC in comparison with ACA or normal adrenal glands [33,34]. *IGF-2* stimulates expression of the ACTH receptor gene (*MC2R*), *CYP17A1* and steroid secretion in H295R cells [35]. The overall risk of ACT and other tumor development in children with BWS is approximately 7% [36,37].

### 1.4. Multiple Endocrine Neoplasia Type 1 (MEN1)

MEN1 is caused by an inactivating mutation in the *menin* gene, a tumor suppressor located on chromosome 11q13. A heterozygous inactivating germline mutation of MEN1 is seen in 90% of families with MEN-1 [38]. The adrenal pathology of MEN1 patients varies, and may result in ACA, bilateral hyperplasia, or ACC in rare cases [39,40]. While somatic mutations of MEN1 are very rare in ACT, LOH at 11q13 has been described in over 90% of informative ACCs and 20% of ACAs [41,42,43].

### 1.5. Wnt Pathway

The Wnt signaling pathway is comprised of a group of highly conserved growth factors that regulate a variety of developmental processes like cell adhesion, proliferation and determination of cell fate, and plays a central role in both development and homeostasis [44]. Members of the Wnt family bind to Frizzled receptors on the cell surface and initiate a signaling cascade resulting in the disruption of a β-catenin degradation complex composed of glycogen synthase kinase 3β (GSK3β), adenomatous polyposis coli (APC), and axin. The net effect of disruption of this complex is stabilization of β-catenin, which translocates to the nucleus to enhance gene expression through interactions with various transcription factors and coactivators [44,45]. Abnormal cytoplasmic and/or nuclear accumulation of β-catenin has been seen in 30% of ACAs and 85% of ACCs. Somatic activating mutations of β-catenin are present in approximately 30% of ACAs and ACCs [46]. This suggests that β-catenin mutation maybe an early step in a common multistep progression of ACA and ACC [46]. The presence of activating β-catenin mutations in ACC was associated with worse prognosis on transcriptome analysis [28].

### 1.6. Carney Complex (CNC) & Isolated Primary Pigmented Nodular Adrenocortical Disease (PPNAD)

Carney Complex is an autosomal dominant disorder that results from inactivating mutations in the protein kinase A regulatory subunity (*PRKAR1A*) gene, that encodes the type 1α regulatory subunit of cAMP dependent PKA. Patients with CNC develop PPNAD with abnormal skin pigmentation, hypercortisolism, cardiac myxomas and other tumors. Isolated PPNAD and CNC have both been associated with inactivating mutations in *PRKAR1A* located on chromosome 17q22-24 [47]. Somatic inactivating mutations or allele losses of *PRKAR1A* locus are also seen in sporadic ACA and ACC [48].

### 1.7. Gardner Syndrome

Gardner syndrome is an autosomal dominant disorder that results from mutations in the *APC* gene on chromosome 5q21. It is associated with the development of multiple colonic adenomatous polyps, and is associated with an increased risk of colorectal cancer. Patients develop adrenal tumors more frequently than the general population, however most adrenal masses are non-functional and benign [49].

## 2. Molecular Markers

Given the diagnostic challenges associated with the definitive diagnosis of ACC, especially in the absence of widely invasive or metastatic disease, identification of molecular markers has been the focus of much recent research. Several types of genomic studies such as gene expression analysis, comprehensive genomic hybridization (CGH), DNA methylation profiling, and miRNA profiling have helped elucidate the roles of several involved genes/pathways that could be used for classification, diagnostic or prognostic purposes. We recently demonstrated that somatic *KCNJ5* mutations occur frequently in aldosterone producing ACA [50], but have not been identified in other ACTs. 

A subject of much study, and debate, has been the possibility of stepwise progression of tumorigenesis, from benign to malignant ACTs. Arguments put forth are the epidemiology of ACTs and the lack of clear evolution from benign adenoma to ACC based on follow up of non-operated tumors [3,51]. However studies suggest that a significant number of distinct genetic alterations in adenomas are also seen in carcinomas, and cases have been reported which demonstrate both benign and malignant pathological areas of the same tumor [52]. Studies utilizing CGH [53] to study ACTs support the theory of progression from adenoma to carcinoma. Sidhu *et al.* suggested that activation of a protooncogene on chromosome 4 may be an early event, with progression from ACA to ACC involving activation of oncogenes on chromosomes 5 and 12 and inactivation of tumor suppressor genes on chromosome arms 1p and 17p [16]. Studies also suggest a positive correlation between the number of CGH alterations and tumor size [16,17]. Additionally studies of ACT clonality also demonstrate that most ACAs and all ACCs consist of a monoclonal population of cell, in contradistinction to hyperplastic adrenal tissue, which is usually polyclonal [54,55,56]. 

## 3. Gene Expression Analysis

Several genome-wide expression studies (Table 1) have been performed over the last decade, which have identified a number of diagnostic markers [5,33,34,57,58,59,60,61,62]. IGF-2 has been consistently exhibited over-expression in a number of studies [33,34,57,58,62,63]. Soon *et al*. also demonstrate that IGF-2 can be used, in combination with Ki-67, with 96% sensitivity and 100% specificity in diagnosing ACCs [62]. IGF-2 is a growth factor that regulates growth and apoptosis through an interaction with the IGF-1 receptor (IGF-IR). Studies of ACT in the pediatric population have also demonstrated overexpression of IGF-2 in ACT compared to normal adrenal tissue, however to a lesser degree of over-expression compared to adult tumors. In addition, the majority of ACTs overexpressed immature forms of IGF-2 [63]. In another study evaluating the expression of IGF-2 in pediatric and adult ACT, IGF-2 was found to be overexpressed in adult ACC, whereas IGF-IR was found to be overexpressed in pediatric ACC. While IGF-IR expression was similar in benign and malignant adult ACT, IGF-IR over-expression was identified only in pediatric adrenocortical carcinomas, and was also found to be a predictor of metastases in children with ACT [64]. Several other growth factors have also been shown to be overexpressed in ACC, including TGFβ2 [34], TGF-β1 [65], insulin like growth factor-related genes; IGF2, IGF2R, IGFBP3 and IGFBP6 [58], and VEGF [66].

**Table 1:** Gene expression profiling studies performed in adrenocortical tumors.

Study	Samples studied	Genes with suggested alteration in expression levels
Giordano TJ (2003)	ACC (N = 11) compared to ACA (N = 4) and normal adrenal cortical tissue (N = 3)	Upregulated: *IGF2*, osteopontin (*SPP*), serine threonine kinase 15 (*STK15*), Angiopoietin 2 (Ang-2), ectodermal-neural cortex one (ENC-1) gene, high mobility group A2 (HMG2) gene in ACC compared to ACA
Velázquez-Fernández D (2005)	ACC (N = 7) compared to ACA (N = 13)	Upregulated: 2 ubiquitin-related genes ( *USP4* and *UFD1L*), insulin like growth factor-related genes (*IGF2*, *IGF2R*, *IGFBP3* and *IGFBP6* in ACC compared to ACA Downregulated: cytokine gene (*CXCL10*), several genes related to cell metabolism (*RARRES2*, *ALDH1A1*, *CYBRD1* and *GSTA4*), and the cadherin 2 gene (*CDH2*) in ACC compared to ACA
De Fraipont F (2005)	ACC (N = 24) compared to ACA (N = 33)	Upregulated: High expression of genes that encode growth factors: (IGF2 and TGFβ2), growth factor receptors: FGFR1, FGFR4, *MST1R*, TGFBR1, KCNQ1OT1 and *GAPDH* in ACC compared to ACA Downregulated: Low expression of steroidogenesis genes: steroidogenic acute regulatory protein (*StAR*), *CYP11A*, HSD3B1, CYP11B1, CYP21A2 and CYP17 in ACA compared to ACC
Slater EP (2006)	ACC (N = 10) compared to ACA (N = 10) and normal adrenocortical tissue (N = 10)	Upregulated: *IGF2* upregulated in ACC compared to ACA Downregulated: *Chromogranin B*, early growth response factor 1 (*EGF1*) downregulated in ACC compared to ACA
West AN (2007)	ACC (N = 18) compared to ACA (N = 5) and normal adrenocortical tissue (N = 7)	Upregulated: Increased gene expression of FGFR4 and IGF2 in ACT compared to normal adrenocortical tissue Downregulated: decrease in KCNQ1, CDKN1C, and HSD3B2 gene expression in ACT compared to normal adrenocortical tissue
Fernandez-Ranvier GG (2008)	ACC (N = 11) compared to ACA (N = 43)	Downregulated: *SERPING1*, *MRPL48*, *TM7SF2*, *DDB1*, *NDUSF8*, *PRDX5* downregulated in ACC compared to ACA
Fernandez-Ranvier GG (2008)	ACC (N = 11) compared to ACA (N = 74)	37 genes differentially expressed between ACC & ACA, 5 of which had high diagnostic accuracy Upregulated: *IL13RA2* and *CCNB2* Downregulated: *HTR2B*, *RARRES2*, and *SLC16A9*
Giordano TJ (2009)	ACC (N = 33) compared to ACA (N = 22) and normal adrenocortical tissue (N = 10)	Cluster analysis of ACCs revealed two different prognostic subtypes that reflected tumor proliferation and survival differences Downregulated: Decreased expression of *NOV* and *NR4A2* in ACC compared to ACA & normal tissue
Soon P (2009)	ACC (N = 12) compared to ACA (N = 16) and normal adrenocortical tissue (N = 6)	Upregulated: *IGF2*, *MAD2L1*, and *CCNB1* in ACC compared to ACA Downregulated: *ABLIM1*, *NAV3*, *SEPT4*, and *RPRM* in ACC compared to ACA A combination of *IGF2* and *Ki-67* has 96% sensitivity and 100% specificity in diagnosing ACC
De Reynies A (2009)	153 ACT	Combined expression of DLG7 (upregulated) and PINK1 (downregulated) was the best predictor of disease-free survival in ACT Combined expression of BUB1B (downregulated) and PINK1 (downregulated) was the best predictor of overall survival in ACT

Szabo *et al.*, in a meta-analysis of gene expression and CGH profiling data, revealed three major pathogenetic pathways to be dysregulated in ACC: cell cycle (c-MYC, CDC25B, CCNB2, CDC2, TOP2A CCNE1, CDK2, CDK7, UBC, MDM-2), retinoic acid signaling (RXRA, ALDH1A1, ALDH1A1-3), and complement system and antigen presentation SERPING1 and MHCII) [67].

Soon *et al.* demonstrate that a combination of IGF2 and Ki-67 exhibit 96% sensitivity and 100% specificity in diagnosing ACCs [62]. The combination of IGF2 and Ki-67 was also shown by Schmitt et al to accurately predict malignancy in ACT [68].

Several studies have evaluated prognostic markers in ACC. In a cluster analysis of the ACCs, Giordano *et al*. revealed two different prognostic subtypes that reflected tumor proliferation and survival differences [61]. In another study, combined expression of DLG7 (overexpressed) and PINK1 (under-expressed) was shown to be the best predictor of disease-free survival in ACT. Combined expression of BUB1B and PINK1 (decreased expression) was the best predictor of overall survival in ACC [5].

Morimoto *et al.* analyzed the Ki67 labeling index, and demonstrated that an index of 7% or more was associated with significantly shortened disease-free survival in ACC [69]. Sbiera *et al*. demonstrated that the SF-1 gene and protein expression significantly correlated with poor clinical outcome, including tumor stage-adjusted hazard ratio for death and recurrence [70]. SF-1 is amplified and overexpressed in childhood ACC [71,72], increases proliferation, and decreases apoptosis of human adrenocortical cells, and induces adrenocortical tumors in transgenic mice [71,72,73].

Two recent studies that classified ACCs according to their transcriptome identified two distinct prognostic groups of ACCs [61,74]. Ragazzon *et al.* describe two prognostic clusters with genes involved in transcription and the cell cycle overexpressed in the poor-outcome group, and genes involving cell metabolism and intracellular transport overexpressed in the good-outcome group [74]. Cluster analysis of the ACCs performed by Giordano et al revealed two subtypes that reflected tumor proliferation, as measured by mitotic counts and cell cycle genes [61].

## 4. Comprehensive Genomic Hybridization (CGH)

ACCs are associated with significant chromosomal gains and losses compared to ACAs, and these changes have been demonstrated to correlate with tumor size [53].

Chromosomal gains in 6q, 7q, 12q, and 19p, and chromosomal losses in 3, 8, 10p, 16q, 17q, and 19q are associated with worse prognosis [53]. Mateo *et al*., in a study on pediatric and adolescent ACTs, also demonstrated a relationship between tumor size and number of chromosomal changes. The most frequent chromosomal changes were gains were of the 1p21-p31.2 and 2p12-p21 regions, and the loss of region 20p11.2-p12, more frequently in ACC compared with ACA, thus supporting the concept of a progressive pattern in adrenocortical tumorigenesis, previously mentioned in other studies on adult ACTs [16,43]. A diagnostic tool developed by Barreau *et al*., by combining DNA copy number estimates at six loci (5q, 7p, 11p, 13q, 16q, and 22q) distinguished ACC from ACA in an independent validation cohort with a sensitivity of 100% and specificity of 83% [75].

## 5. DNA Methylation Profiling

There is a growing body of evidence to suggest that epigenetic abnormalities including DNA methylation and histone modification play a key role, in conjunction with genetic modifications, to cause altered patterns of gene expression, resulting in tumorigenesis. DNA hypermethylation of the promoter cytosine in cytosine phosphate guanine islands (CpG islands) causing downstream gene silencing has been shown to be intimately involved in tumorigenesis [76,77,78]. Altered DNA methylation of the H19 promoter has been shown to be involved in the abnormal expression of both *H19* and *IGF-2* genes in adrenocortical carcinomas [79]. Promoter methylation of *TP53* however, has been demonstrated not to be a significant event in the development of adrenocortical carcinomas [80]. Two recent genome-wide DNA methylation profiling studies have shed additional light on the role of methylation in ACTs. We described six genes (CDKN2A, GATA4, DLEC1, HDAC10, PYCARD, SCGB3A1/HIN1) that were demonstrated to be significantly hypermethylated in ACC when compared with ACA and normal tissue. This finding was also associated with a corresponding decrease in gene expression. In addition, treatment of H295R cells with a demethylating agent restored expression of the hypermethylated genes, indicating that this is a reversible event [13]. Rechache *et al*. demonstrate that ACC samples were globally hypomethylated and describe distinct methylation patterns that could distinguish normal, benign, primary malignant, and metastatic tissue samples [81].

## 6. MicroRNA Profiling

MicroRNAs (miRNAs) are short non-coding RNAs that are involved in post-transcriptional regulation of gene expression. miRNAs regulate approximately one third of coding genes, therefore changes in miRNA expression may be associated with cancer development and progression.

miR-483-5p is one of the most investigated miRNAs involved in ACC. It has been shown to be upregulated in ACC compared to ACA, and has been identified as a predictor of poor prognosis in ACC [14,15]. miR-483-5p and IGF2 were also demonstrated to be statistically significantly co-expressed in ACC [14]. Given that miR-483-5p is located at 11p15.5 within the second intron of IGF2, it is likely that increased expression of miR-483-5p observed in ACC may be an indirect consequence of IGF2 over-expression. miR-483-3p was also found to be upregulated in ACC compared to ACA, which significantly correlated with miR-483-5p levels [82]. This upregulation of miR-483-3p was also seen in the study by Soon *et al.* [15], as well as in pediatric ACT [83]. miR-503 was also found to be upregulated in ACCs compared to normal adenocortical tissue and ACA in both pediatric and adult ACTs [15,83].

miR-184 has also been shown to be over-expressed in ACCs compared to ACAs [84]. miR-195 is another miRNA that has been the focus of much study in ACC. It is significantly down-regulated in ACCs compared to ACAs, and its low expression in ACCs is significantly associated with poor overall disease specific survival [14,15], It is also down-regulated in childhood ACTs [83]. 

miR-7 and miR-335 are also significantly down-regulated in ACCs compared to ACAs and normal adrenocortical tissue [15]. miR-214 and miR-375 are similarly significantly down-regulated in ACCs compared to ACAs and normal adrenocortical tissue in both adult and pediatric ACT [83,84].

## 7. Conclusions

Given the lack of a definitive histopathologic method of diagnosis of ACC, especially in the absence of widely invasive or metastatic disease, the identification of molecular markers has been the focus of much recent research. Mutations found in familial cancer syndromes have contributed significantly to our understanding of the pathogenesis of sporadic ACTs. In addition, genome-wide gene expression and DNA methylation studies, along with miRNA profiling have helped identify certain diagnostic and prognostic markers. There are however, substantial differences in the candidate molecular markers identified, indicating a heterogenous genetic and epigenetic basis. This, together with the relative rarity of ACC and the resultant small sample sizes analyzed, signify that additional studies are still required to further our understanding of this disease process.
